# Toward a public health leadership national training agenda: a review of conceptual frameworks and core competencies

**DOI:** 10.3389/fpubh.2025.1630046

**Published:** 2025-08-07

**Authors:** Emily M. Burke, Jordan A. Fox, Kaitlin Tager, Sara McDowell, Fawn Phelps, Howard Koh

**Affiliations:** ^1^Association of Schools and Programs of Public Health, Washington, DC, United States; ^2^Harvard T.H. Chan School of Public Health, Boston, MA, United States

**Keywords:** academic public health, public health leadership, leadership competencies, leadership framework, public health leadership training

## Abstract

Strong and effective leadership is essential for the success of public health systems. It serves as the driving force that inspires, guides, and empowers individuals to improve the health of their communities and strengthen their organizations. Leadership is not merely supplementary but a core element in tackling the increasingly complex challenges facing public health today. The COVID-19 pandemic exposed significant weaknesses in our systems for emergency preparedness and response, highlighting just how critical capable leadership is within governmental public health. These challenges are magnified by persistent workforce issues, including knowledge gaps, limited development opportunities, and concerns around long-term sustainability. Further complicating the landscape are rising levels of political polarization and incidents of harassment directed at public health professionals. In response to these pressures and a growing wave of workforce attrition, there is now a heightened national focus on developing the next generation of public health leaders. This development must be intentional and structured, relying on well-designed, competency-based approaches rather than informal or inconsistent methods. Public health leaders must be equipped with the skills to navigate the evolving demands of modern health systems. In 2022, the Association of Schools and Programs of Public Health (ASPPH) convened an expert panel to develop a national leadership training agenda tailored to the governmental public health workforce. The panel consisted of 15 academic and practice leaders in the United States. Between 2022 and 2024, the panel met regularly to define the essential attributes of public health leadership and determine effective strategies for cultivating them through education and capacity-building efforts. The result of this collaborative effort is the ASPPH Public Health Leadership Competency Mapping and Training Agenda: a foundational framework designed to strengthen capabilities across current and future governmental public health professionals. This article presents that framework, marking an important step toward building a more resilient, competent, and adaptive public health workforce.

## 1 Background

In today’s rapidly evolving public health landscape, strong and adaptable leadership is more critical than ever. Public health professionals – regardless of their role or setting – must be prepared to take on leadership responsibilities. Workforce pressures, such as high turnover, retirement, and growing demands, have intensified the need for leadership preparation across all levels of the field (1). Research shows that governmental public health leaders often serve short tenures, with a median of just 3 years (2). Early exposure to leadership concepts is valuable, but delivering such education remains challenging due to the public health workforce’s decentralized and interdisciplinary nature (3, 4). Compounding this issue is the fact that only a small proportion of the workforce – just 14% – holds a formal degree in public health (3–5).

While the Council on Education for Public Health (CEPH) rightly includes leadership as a foundational competency for both Master of Public Health (MPH) and Doctor of Public Health (DrPH) programs (6), implementation of this competency across curricula is often uneven, and limited opportunities exist to reinforce leadership development through applied practice. As such, there remains a significant need to strengthen and systematize the integration of leadership training throughout the education-to-practice pathway.

Despite the proliferation of frameworks and initiatives, public health leadership development remains fragmented, lacking a unified direction (7). Often, individuals seeking specialized public health leadership training must look to business or corporate leadership frameworks, which may not translate well to the values, contexts, and collective priorities that define public health practice (8). Public health challenges are often enormous in scale, impact populations broadly, and regularly fall outside a single authority’s control. This context demands leadership approaches that are collaborative and oriented toward systems change.

Public health leadership is often taught theoretically, but theory alone will not ready students and practitioners for leadership roles in today’s increasingly challenging public health landscape (7, 9, 10). To truly prepare them, leadership education must bridge theory with practice, equipping learners with both a conceptual understanding and concrete skills to lead effectively in diverse, uncertain, volatile, and high-stakes environments.

To address these challenges, the Association of Schools and Programs of Public Health (ASPPH) convened a national expert panel of academic and practice leaders to define core components of public health leadership and determine how the profession can better cultivate future leaders. This effort culminated in the creation of a national leadership training agenda specifically tailored for the governmental public health workforce. We present a detailed examination of the process and its resulting outcomes.

## 2 Methods

To guide this initiative, the authors and expert panel pursued three primary objectives: to identify gaps in existing public health leadership models, develop a guiding framework to address those gaps, and create a training agenda tailored to the governmental public health workforce. To achieve the first objective, the team conducted a comprehensive literature review of peer-reviewed and grey literature, focusing on public health education and competency frameworks. To develop the guiding framework, the expert panel systematically analyzed and synthesized existing models, ultimately adapting and expanding the 3P Framework for Social Innovators into a public health-specific model – the 4P Framework of Public Health Leadership. Finally, to develop the training agenda, the team conducted an environmental scan of leadership-related standards, competencies, and job task analyses, which were then coded, categorized, and mapped to the domains of the 4P Framework to produce a streamlined and practice-oriented capacity-building guide.

### 2.1 Identifying gaps in existing public health leadership models

The authors began this initiative with a thorough literature review. They searched for English-language articles on public health leadership training and education, published between 1988 and 2023, across peer-reviewed journals and grey literature originating from academic and practice-based sources within the United States. They also authors examined websites of relevant organizations, including the Public Health Accreditation Board (phaboard.org) and the Council on Education for Public Health (ceph.org). They used the following search terms: public health leadership development, future public health leaders, public health leadership framework, public health leadership, leadership competency framework, public health leadership education, public health leadership initiatives, health leadership competency framework, and public health leadership competency set.

### 2.2 Developing a guiding framework

In parallel with the broader literature review, the authors and expert panel conducted a targeted analysis of existing leadership competency frameworks. This phase aimed to extract key domains, models, and competencies to inform the development of a new guiding framework tailored to the evolving needs of the public health workforce.

They examined several models, including the National Public Health Leadership Development Network (NLN)‘s Public Health Leadership Competency Framework, which categorized 80 competencies into transformational, political, trans-organizational, and team-building domains to guide curriculum development (11, 12); the Council on Linkages Between Academia and Public Health Practice (COL)‘s Core Competencies for Public Health Professionals, which assigns leadership systems thinking skills across three tiers of public health practice (13); and Begun and Malcolm’s Competency Framework, which integrates Service, Adaptive, Integrative, and Complexity Leadership models to define five domains of public health leadership capacity (12). Additional frameworks – such as those developed by Grimm et al. (14), the Public Health Accreditation Board (PHAB)‘s Standards and Measures (15), and collaborative initiatives like the Turning Point Leadership Development National Excellence Collaborative (16)—further emphasized critical leadership knowledge, skills, and applied strategies necessary for effective practice.

While these frameworks contributed valuable perspectives, few provided an integrated structure bridging individual, organizational, and systems-level competencies in a way that is both adaptable and practice-oriented. To address this gap, the expert panel identified the 3P Framework for Social Innovators as a flexible and action-oriented model well suited to the interdisciplinary and collaborative nature of public health leadership (15). The 3P Framework for Social Innovators – consisting of the domains “Problem,” “Person,” and “Pathway” – was formulated by researchers at the Harvard Kennedy School’s Social Innovation and Change Initiative, and provides a strong foundation for organizing leadership competencies across various roles and contexts.

**Figure 1:**
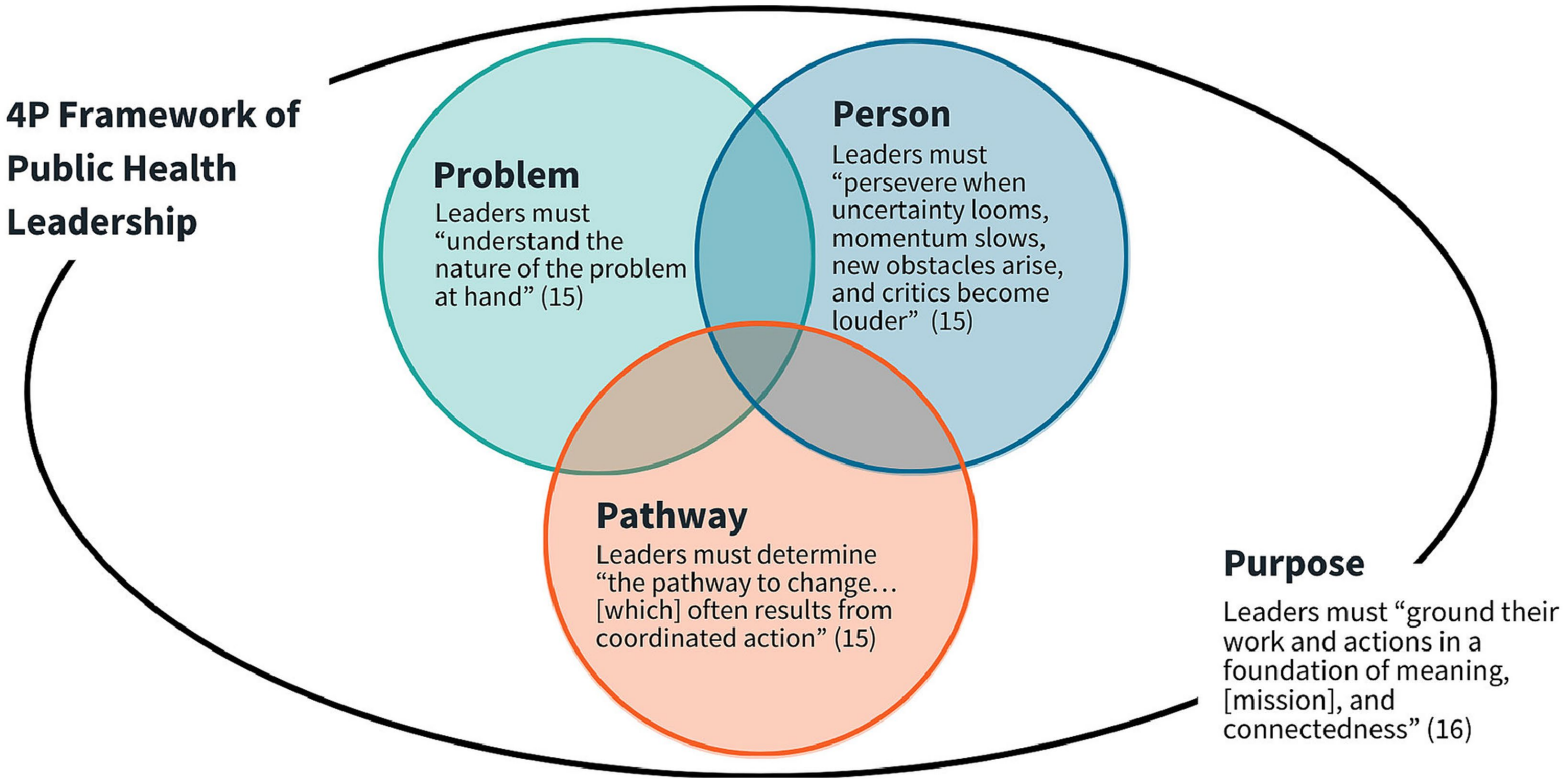
4P Framework of Public Health Leadership.

Acknowledging the deep sense of purpose that defines the public health field, the panel introduced “Purpose” as a fourth domain to elevate the field’s core values and its commitment to equity. This addition led to the development of the 4P Framework of Public Health Leadership. Rooted in both theory and applied leadership practice, the 4P Framework embodies the interdisciplinary, adaptive, and collaborative nature of public health (Figure 1).

### 2.3 Creating a training agenda

Following the development of the 4P Framework, ASPPH and the expert panel conducted a comprehensive environmental scan to examine publicly available public health leadership competencies, strategic frameworks, accreditation measures, and job task analyses. This review aimed to identify the essential needs, requirements, skill sets, and competencies (hereafter collectively referred to as “competencies”) that are critical for strengthening leadership skills in both current and future public health practitioners.

**Figure 2:**
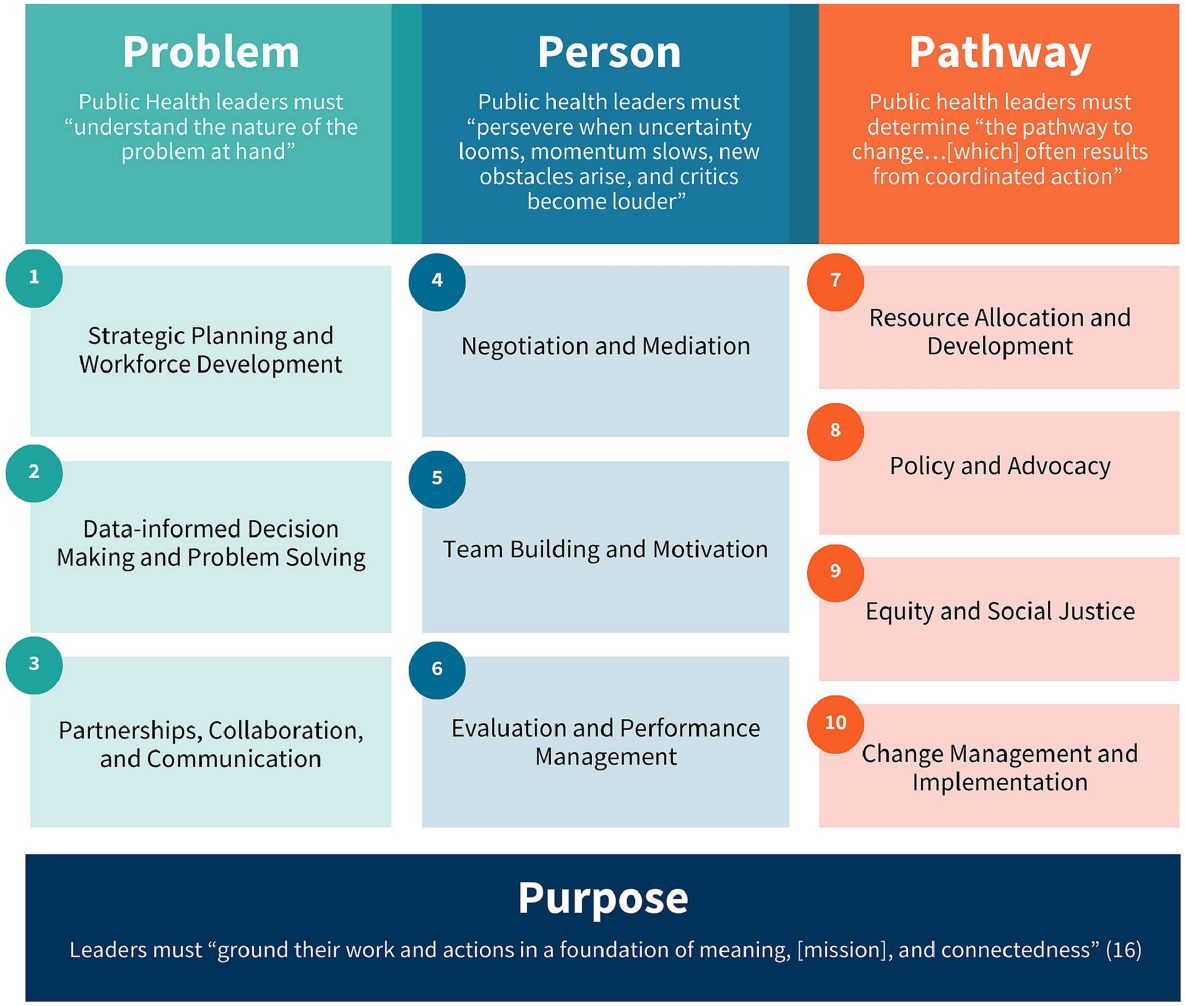
Ten themes/areas identified and segmented into the 4P Framework of Public Health Leadership.

The environmental scan identified several public health leadership competencies. These competencies were consolidated into ten thematic areas aligned with the 4P Framework (Figure 2). This consolidation acknowledges the richness of existing scholarship while addressing the challenge posed by the overwhelming volume and complexity of previously proposed competencies. The examination drew from a wide array of authoritative sources, including the Public Health Accreditation Board’s Standards and Measures (15), the Council on Education for Public Health’s 2021 Accreditation Criteria (6), and the National Board of Public Health Examiners’ 2016 Job Task Analysis (17).

Keyword searches were systematically conducted across all source documents focusing on leadership terms. Competencies containing at least one relevant keyword such as “leadership,” were extracted and cataloged into a structured spreadsheet. For each identified competency associated information was recorded including competency descriptions relevant knowledge and skills and their applicability to public health leadership. In addition to keyword-based extraction all documents were manually reviewed to identify additional competencies that while not containing specific keywords were nonetheless relevant to leadership in public health contexts. It is noteworthy that several competency frameworks contained no explicit references to leadership.

Once leadership-related competencies were identified, they were analyzed and grouped into thematic areas to better reflect the core domains of public health leadership. Many competencies intersected multiple themes, highlighting the interdisciplinary and cross-cutting nature of leadership in public health. After categorization, each competency was entered into a master database that captured key contextual and organizational details. This database included the full competency statement, assigned thematic categories, source organization, and relevant tags for classification. Descriptive annotations were added to provide context – such as how and where the competency was developed – along with direct links to the original framework documents. This structure supports transparency, consistency, and ease of use for training design and future research.

## 3 Results

### 3.1 Gaps in existing public health leadership models

The literature review yielded 79 relevant publications, documents, and webpages related to public health leadership training and education. These included peer-reviewed articles, grey literature, and online resources published in the United States between 1988 and 2023. Three primary themes emerged from this review: leadership frameworks and competency models; core knowledge, skills, and abilities for public health leaders; instructional modalities used in leadership education.

Findings confirmed that leadership is widely recognized as a cornerstone of effective public health practice. Many sources cited the 1988 Institute of Medicine (IOM) report, *The Future of Public Health*, as a catalyst for formal leadership development efforts (18). However, despite decades of activity in this area, key gaps persist. The literature revealed a fragmented landscape in which many competency models emphasize either individual or organizational capacities but rarely bridge the full spectrum from personal to systems-level leadership. Additionally, public health leadership frameworks and initiatives vary considerably in content, rigor, and instructional format, often lacking scalability or alignment with evolving public health challenges.

These insights underscored the need for a streamlined, integrated, and practice-informed model of leadership that could accommodate the interdisciplinary, collaborative, and equity-driven nature of the field.

### 3.2 A guiding framework

Analysis of existing public health leadership models and frameworks revealed common elements such as collaboration, systems-thinking, adaptability, but also highlighted persistent limitations. Many lacked explicit alignment with core values like equity, or failed to integrate personal, organizational, and systems-level competencies into a cohesive structure. In response, as mentioned above, the expert panel identified the 3P Framework for Social Innovators.

Social innovation is defined as the creation of ideas, products, and services that tackle environmental and social challenges (19). The 3P Framework is a reflective tool to help social innovators align personal motivations with systemic challenges and strategic approaches to effect meaningful change (20). In this context, “Problem” emphasizes the importance of deeply understanding the social issue at hand; “Person” focuses on the individual change agent, including their values and experiences; and “Pathway” involves mapping out a strategic route to enact change, including identifying actionable steps and considering a solution’s sustainability (20). To tailor the 3P model to the unique demands of public health, the panel introduced the fourth domain, “Purpose,” which centers public health’s mission-driven ethos and elevates values such as service, justice, and community engagement.

The result is the 4P Framework of Public Health Leadership, which synthesizes insights from existing models while responding to the field’s practical and moral imperatives. Building on the original 3P Framework, the expanded 4P model guides leaders in exploring problems, crafting solutions, and driving innovation, with a sustained sense of purpose and vocation permeating all aspects of leadership. This focus on exploring strengths, weaknesses, values, and motivations forms the foundation for developing personal and systemic mastery and fostering a deep connection to public health work (21). Such mastery offers meaning in one’s career and empowers public health leaders to guide teams, organizations, and systems, even during times of volatility and uncertainty. By promoting an iterative approach, the 4P Framework of Public Health Leadership encourages leaders to continually reassess their assumptions and strategies in response to changing conditions. It emphasizes flexibility, reflection, and responsiveness in addressing complex public health challenges

### 3.3 Mapping competencies to the 4P framework

**Figure 3:**
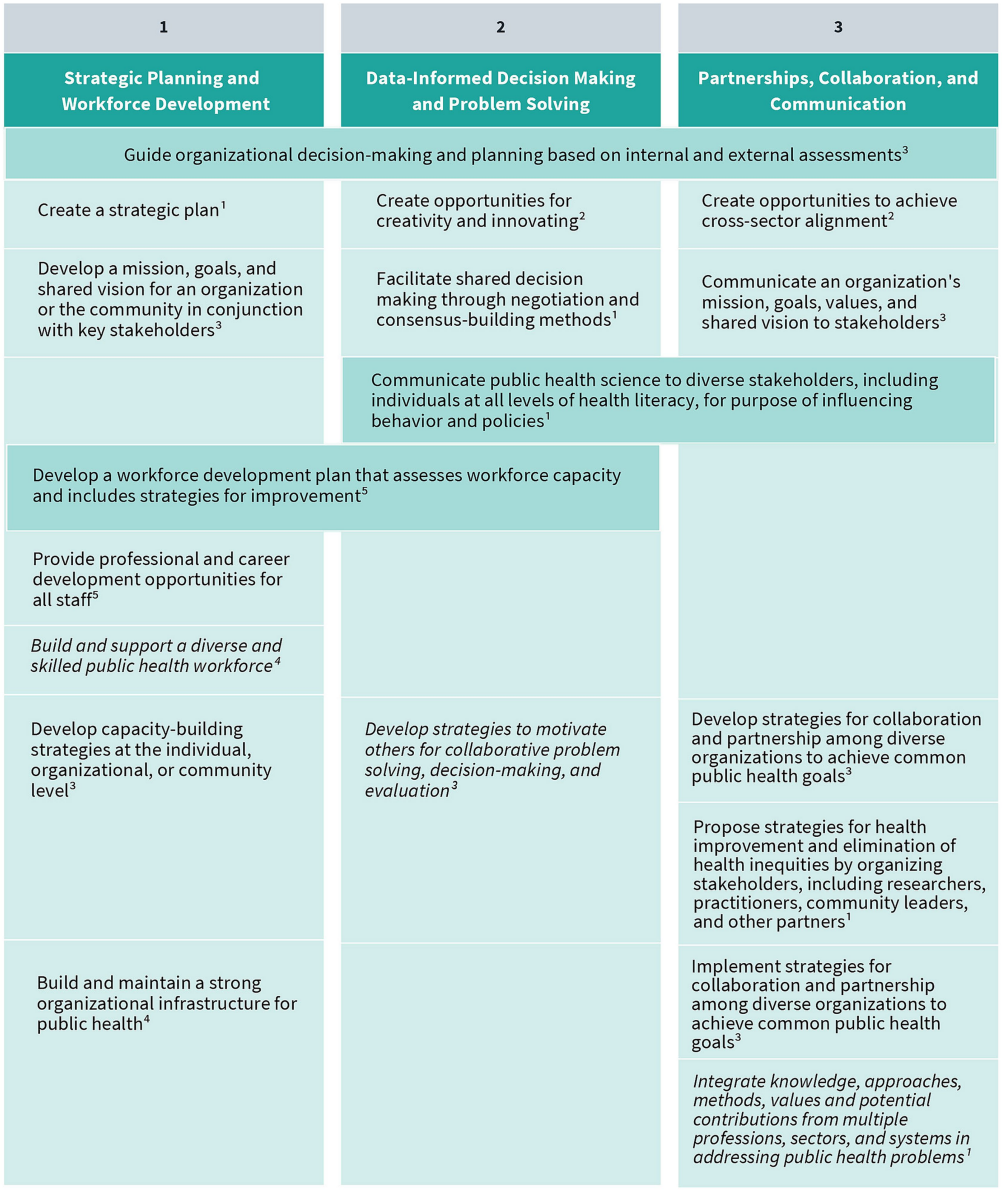
Strategic planning and workforce development, data-informed decision making and problem solving, and partnerships, collaboration, and communication competencies. Competencies that fall under multiple key themes are merged horizontally across the table. Italicized text indicates that a competency belongs to multiple “P’s” in the 4P Framework. (1) CEPH; (2) COL; (3) NBPHE; (4) PHAB Center for Innovation; (5) PHAB Standards and Measures.

**Figure 4:**
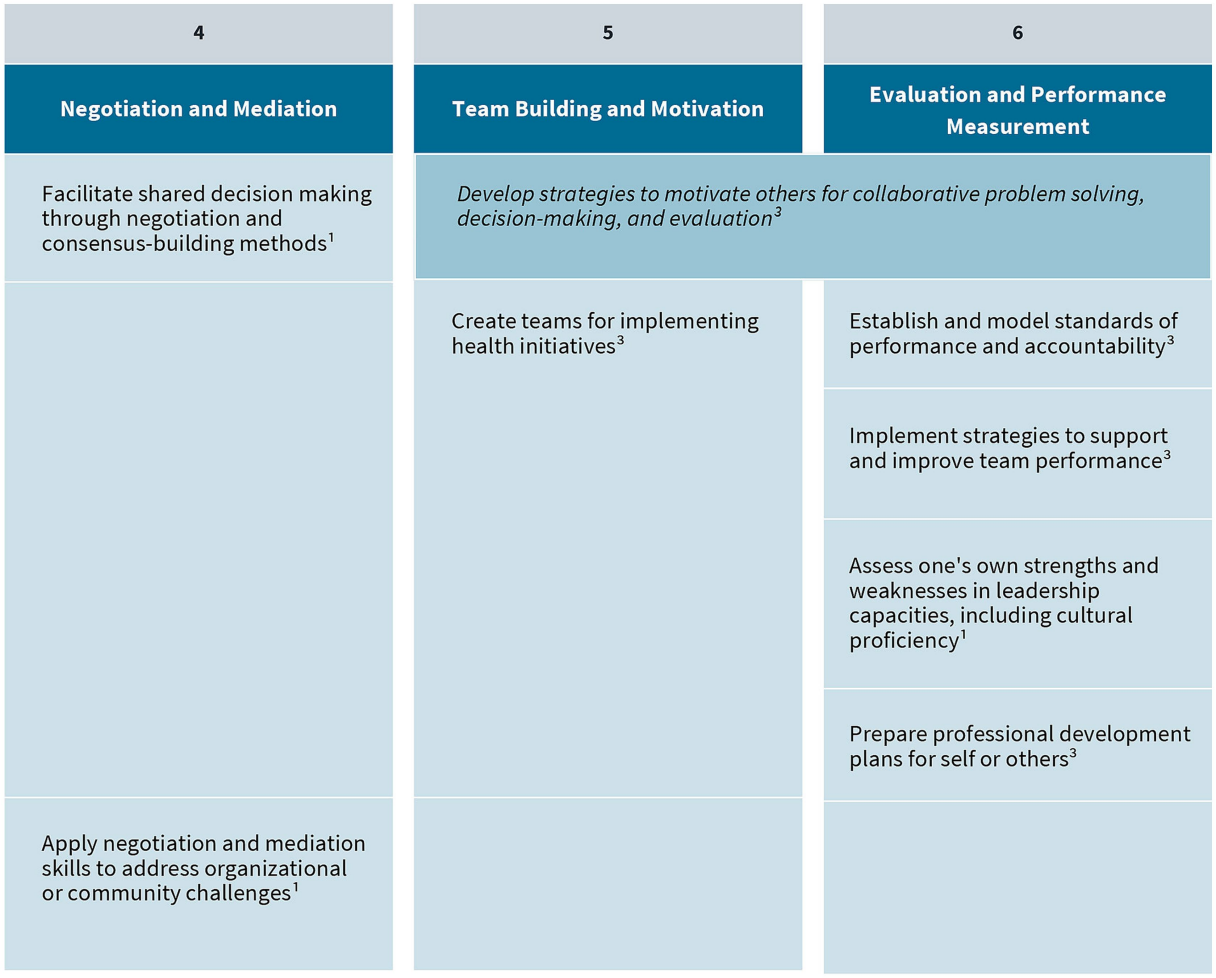
Negotiation and mediation, team building and motivation, and evaluation and performance management competencies. Competencies that fall under multiple key themes are merged horizontally across the table. Italicized text indicates that a competency belongs to multiple “P’s” in the 4P Framework. (1) CEPH; (2) COL; (3) NBPHE; (4) PHAB Center for Innovation; (5) PHAB Standards and Measures.

**Figure 5:**
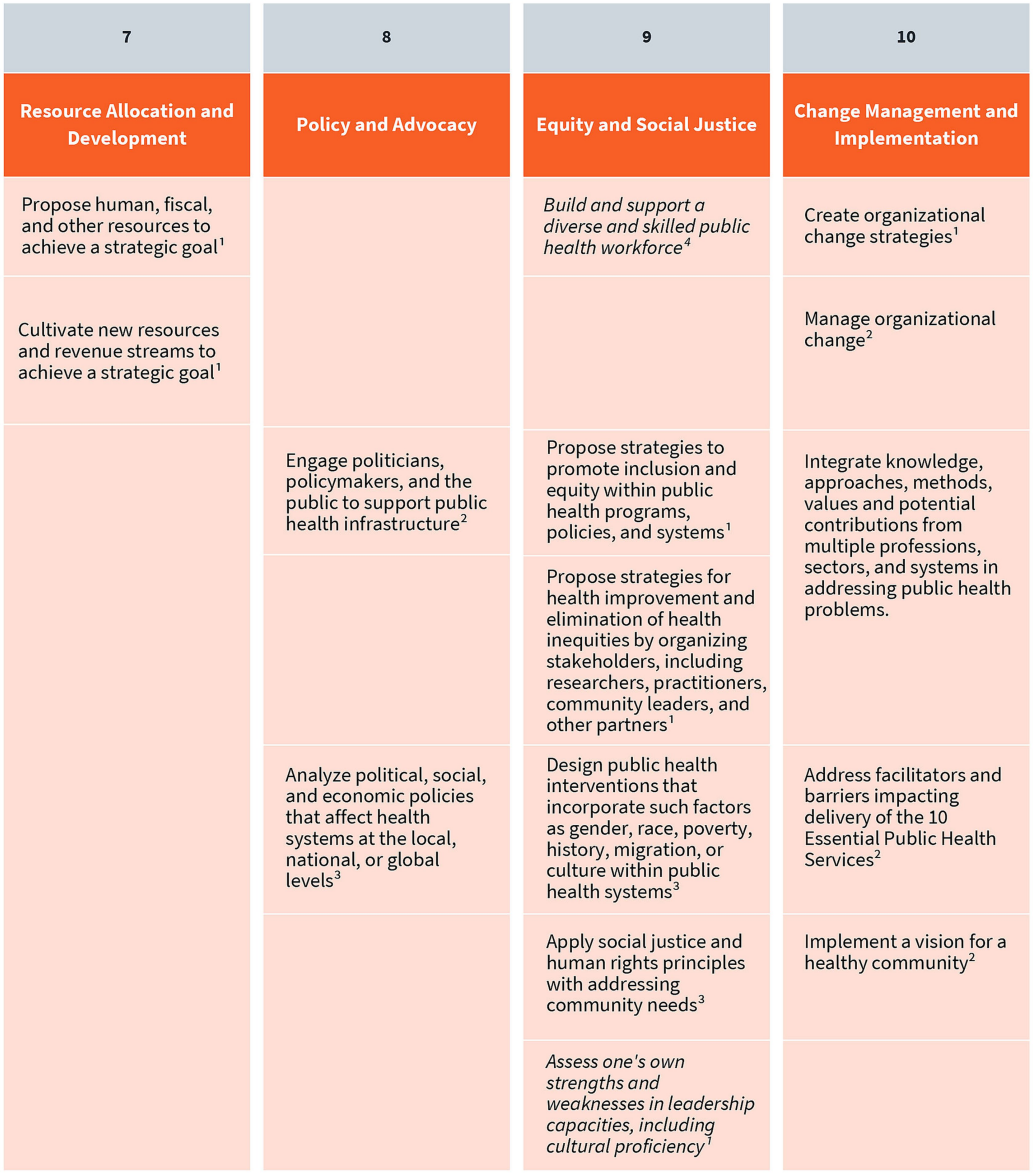
Resource allocation and development, policy and advocacy, equity and social justice, and change management and implementation competencies. Competencies that fall under multiple key themes are merged horizontally across the table. Italicized text indicates that a competency belongs to multiple “P’s” in the 4P Framework. (1) CEPH; (2) COL; (3) NBPHE; (4) PHAB Center for Innovation; (5) PHAB Standards and Measures.

As a result of their extensive environmental scan, the authors and panel identified a total of 41 public health leadership competencies (visually represented in Figures 3–5). These competencies are organized vertically within each figure according to thematic areas relevant to public health leadership training. To reflect the interdisciplinary nature of leadership skills, competencies that apply to more than one thematic area are displayed horizontally across multiple columns. Additionally, competencies that align with more than one domain of the 4P Framework – “Problem,” “Person,” “Pathway,” and “Purpose” – are italicized to signal their cross-cutting relevance. Superscripts are used throughout the figures to denote the original source of each competency, drawing from five major frameworks and standards in the field. These sources include:

1. The Council on Education for Public Health (CEPH)‘s 2021 Accreditation Criteria, which outline the foundational competencies required for the accreditation of public health programs and degrees at the bachelor’s, MPH, and DrPH levels (6);
2. The Core Competencies for Public Health Professionals, developed by the Public Health Foundation’s Council on Linkages Between Academia and Public Health Practice, which provide a consensus-based foundation of cross-cutting knowledge and skills for public health practice, education, and research (22);
3. The 2016 Job Task Analysis from the National Board of Public Health Examiners, which identifies and categorizes the essential tasks performed by public health professionals (17);
4. The 10 Essential Public Health Services, revised in 2020 by the Public Health Accreditation Board (PHAB)‘s Center for Innovation, which emphasize policy, systems change, and equity as the foundation of public health service delivery (23); and
5. PHAB’s Standards and Measures for Initial Accreditation, which offer a comprehensive framework for evaluating and guiding the performance of health departments seeking national accreditation (15).

Together, these sources provided the foundational material for mapping leadership competencies across the 4P Framework, ensuring that the resulting agenda is grounded in recognized standards and relevant to the diverse roles within the public health workforce. The competency mapping not only reinforces the relevance of established public health standards but also enables a structured analysis of how leadership capacities are distributed across the domains of the 4P Framework. In doing so, it facilitates a nuanced understanding of the specific cognitive, interpersonal, and strategic demands associated with each domain.

#### 3.3.1 Problem

The “Problem” domain of the 4P Framework emphasizes the importance of deeply understanding the complex challenges public health leaders must confront. Effective leadership in this domain begins with the ability to deeply examine a problem’s context, including contributing systemic factors and the perspectives of affected communities and partners. Rather than rushing to solutions, leaders are encouraged to approach problems with curiosity, empathy, and analytical rigor. Competencies such as strategic planning, workforce development, and data-informed decision making are essential here, as they allow leaders to frame problems accurately and develop responsive, equity-oriented strategies. In addition, cultivating skills in communication and cross-sector collaboration ensures that problem-solving is not done in isolation but in concert with diverse stakeholders.

#### 3.3.2 Person

The “Person” domain focuses on the inner dimension of leadership: self-awareness, motivation, and the ability to influence others. Public health leaders must understand their own leadership styles, values, and positional power, whether formal or informal. Effective leadership in this domain requires the capacity to build trust, mobilize teams, and align people toward shared goals. Competencies such as negotiation and mediation, team building and motivation, and performance management are central. These capabilities allow leaders to navigate interpersonal dynamics, foster cohesion, and sustain engagement in complex or high-pressure environments. By investing in their own development and cultivating relational intelligence, leaders strengthen their ability to lead with authenticity and impact.

#### 3.3.3 Pathway

The “Pathway” domain addresses the ways leaders move from intention to action, or how they implement change, influence systems, and ensure sustainability. It emphasizes the importance of identifying multiple avenues for change, including direct leadership, collaboration, and alignment with broader institutional and community structures. Competencies relevant to this domain include resource allocation and development, policy and advocacy, equity and social justice, and change management. These areas equip leaders to address systemic barriers, shift organizational practices, and build coalitions that support long-term impact. The Pathway domain reflects a pragmatic understanding that leadership is not a solo endeavor but a strategic, adaptive process that leverages relationships, resources, and context to drive meaningful results.

#### 3.3.4 Purpose

The “Purpose” domain serves as the foundation of the 4P Framework, grounding leadership in meaning, mission, and connectedness. This dimension draws from the concept of spiritual or values-based leadership, not in a religious sense, but in the sense of being guided by a connection to a cause greater than oneself (21). Spiritual leadership, an adaptation of values-based leadership, is defined as the internal values, attitudes, and behaviors that drive intrinsic motivation in oneself and others, cultivating spiritual endurance through a shared sense of purpose and community (24). This framing highlights the inner motivation and belonging in sustaining leaders through challenges and in enabling them to inspire others with authenticity and conviction. Although public health literature offers few defined competencies in this area, the expert panel and authors adapted guidance from leadership scholarship in other sectors. These include practices such as cultivating mindfulness and reflection, promoting staff well-being and organizational integrity, and fostering inclusive, values-driven environments. Purpose is not easily taught, but it can be nurtured. When it is, it fuels more resilient, compassionate, and visionary public health leaders.

### 3.4 Proposing a national public health leadership training agenda

The 4P Framework of Public Health Leadership provides a structured approach for identifying, organizing, and applying leadership competencies, effectively bridging theory and practice. Using this framework as a guide, the authors developed the Public Health Leadership Competency Mapping and Training Agenda to address gaps in leadership development across the governmental public health workforce. This agenda translates broad leadership concepts into clearly defined, actionable skills that support practical instruction, targeted assessment, and measurable learning outcomes.

The Public Health Leadership Competency Mapping and Training Agenda (“Training Agenda”) addresses a key workforce gap: many public health professionals lack formal public health education or leadership training. This contributes to uneven leadership capacity across agencies and jurisdictions. To help close this gap, ASPPH developed a competency-based instructional model that provides all public health professionals – regardless of background – with access to clear leadership expectations, development pathways, and practical skill-building resources. Grounded in evidence and practice, the agenda offers a scalable model for strengthening leadership throughout the public health system.

Designed to be scalable and adaptable, the Training Agenda equips public health professionals with the competencies needed to lead in diverse and evolving contexts. It supports leadership development at all levels and is intended for use by academic public health programs and governmental employers, including state, Tribal, local, and territorial health departments. By grounding the agenda in both evidence and practice, this initiative aims to build a more resilient, agile, and future-ready public health workforce.

The competency mapping process directly informed the creation of the Training Agenda, designed to address evolving needs and trends in leadership advancement within the governmental public health workforce. Through a structured analysis of existing frameworks, the expert panel and authors identified both areas of alignment and critical gaps, revealing opportunities for the creation of targeted training programs linked to key leadership themes and competencies. To be effective, future academic and professional capacity-building initiatives must be responsive to the multilevel demands of the public health ecosystem, supporting leadership readiness at the individual, organizational, and community levels. Instruction should be grounded in systems thinking, which emphasizes the interdependence of actors, structures, and social determinants within public health systems, and prepares practitioners to navigate complexity, uncertainty, and cross-sector collaboration. Applied learning modalities such as case-based simulations, scenario planning, peer learning cohorts, mentoring programs, and problem-based workshops can bridge the gap between theoretical knowledge and practice. These approaches not only reinforce core competencies but also foster adaptive, collaborative, and equity-driven leadership essential for advancing public health in diverse and rapidly changing environments.

**Figure 6:**
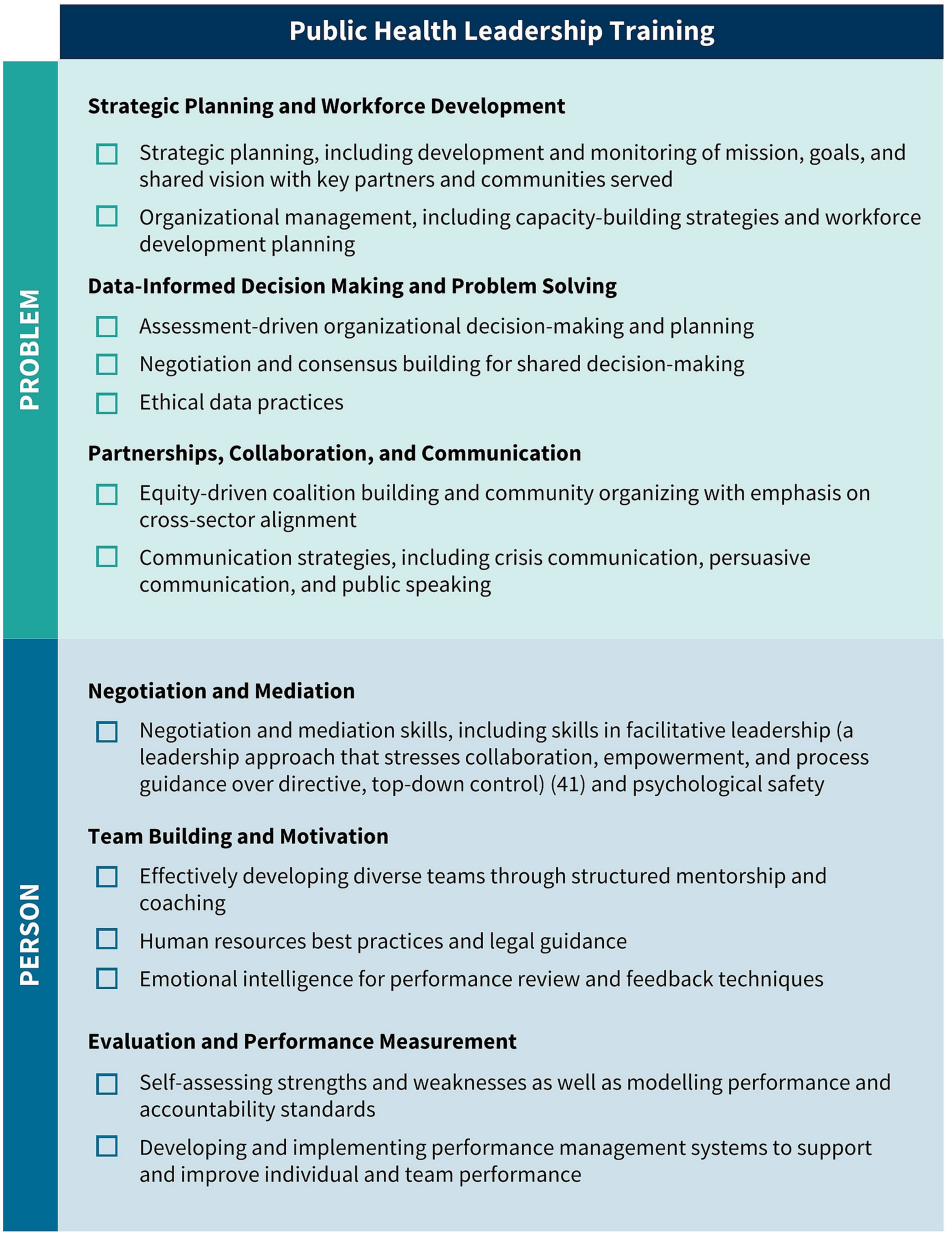
National public health leadership training agenda, part 1.

**Figure 7:**
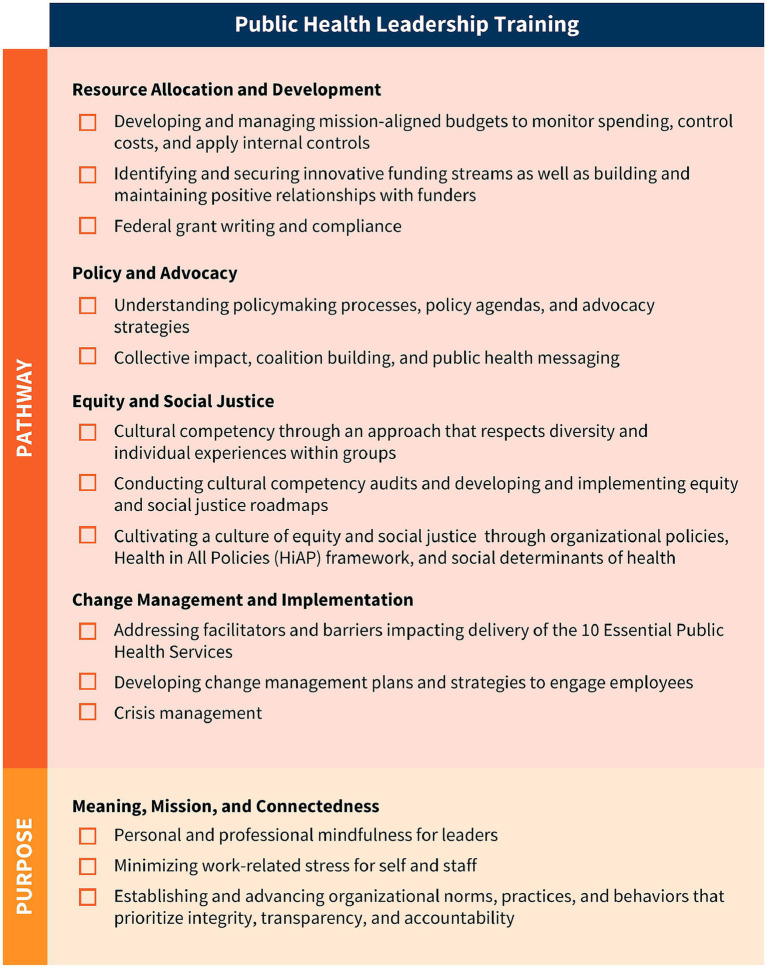
National public health leadership training agenda, part 2.

The Public Health Leadership Competency Mapping and Training Agenda is a tool for action. It is intended to guide both academic institutions offering public health degrees and certificates, and governmental public health employers – including state, Tribal, local, and territorial health departments – seeking to strengthen leadership expertise within their jurisdictions. By providing a shared language and structure for leadership development, the agenda supports the creation of aligned, effective, and scalable strategies to build the next generation of public health leaders. Importantly, the competencies apply to leaders with both formal authority or informal influence – recognizing that public health leadership spans all levels and roles (Figures 6, 7).

## 4 Discussion

Although there is growing recognition of the importance of leadership in advancing public health goals, the field has thus far lacked a cohesive, practice-informed, and actionable framework to guide leadership development across both academic and workforce settings. Existing leadership preparation opportunities are often fragmented, discipline-specific, and misaligned with the complex challenges public health professionals routinely face. Further, the absence of a unifying competency set tailored to public health leadership has hindered the ability of educators, employers, and policymakers to coordinate efforts, assess preparedness, and scale impact; this ultimately limits the field’s capacity to build and sustain public trust in times of both stability and crisis.

Developing a resilient and effective public health workforce demands intentional, sustained investment in leadership education. Given the rising complexity, urgency, and visibility of today’s public health challenges, developing a workforce skilled in adaptive leadership is an imperative, not an option. Cultivating these leadership skills empowers public health professionals to drive strategy, build partnerships, enhance resilience, and maintain public trust through both crisis and recovery.

Current literature has revealed both achievements and persistent gaps in the present landscape of public health leadership education and training. While numerous frameworks and initiatives have contributed to progress, the field still lacks a unified, competency-based, and practice-oriented approach that bridges academic theory with the dynamic demands of real-world public health practice. Too often, leadership development in public health remains siloed, optional, or overly theoretical, and falls short of preparing practitioners for complex realities on the ground.

The Public Health Leadership Competency Mapping and Training Agenda, grounded in the 4P Framework of Public Health Leadership, addresses these gaps and offers a practical and timely response to these challenges. By distilling an expansive array of proposed competencies into a streamlined set of ten thematic areas, the 4P Framework creates a coherent and feasible foundation for embedding leadership development across the public health education-to-workforce pathway. It also provides a shared language and actionable structure for aligning curricula, assessments, and professional training programs with the leadership capabilities most essential to contemporary practice.

At its core, the 4P Framework integrates both personal and systemic dimensions of leadership, encouraging self-reflection, skill-building, systems thinking, and sense of purpose. This holistic approach recognizes that leadership can and must happen at all workforce levels, whether through formal authority or informal influence. The Public Health Leadership Competency Mapping and Training Agenda then translates this framework into actionable learning pathways that support leadership readiness across roles and settings. It is designed to equip public health professionals with the practical competencies needed to lead effectively in dynamic environments, foster collaboration, and drive equity-centered change.

## 5 Conclusion and recommendations

Looking ahead, public health institutions – academic programs, governmental agencies, and professional associations – must elevate leadership development as a central education and workforce priority. This includes embedding leadership training across disciplines and specialties, incorporating it throughout all career stages, and ensuring its adaptability to various learning needs and evolving field realities. A culture of continuous leadership skill-building must be fostered, wherein competencies are regularly refined and grounded in real-world practice.

To fully realize the vision of the 4P Framework and meet the urgent need for public health leadership, the authors offer the following recommendations:

1. Integrate leadership development across all education and practice levels. Leadership skill-building must be a core component of public health education. Public health schools and programs should align curricula with the 4P Framework and embed leadership competencies into undergraduate, graduate, and doctoral-level learning pathways.
2. Ensure leadership preparation is practice-oriented and adaptive. Instructional approaches must reflect the realities of public health work, incorporating experiential learning modalities such as simulations, applied projects, case-based learning, and interdisciplinary collaboration. These methods foster the adaptive skills needed for high-pressure and uncertain environments.
3. Tailor learning opportunities to meet the needs of a multi-tiered workforce. The leadership training agenda must recognize the unique learning needs of professionals at different career stages - entry-level staff, mid-career managers, and executive leaders - while also supporting informal leaders working across sectors.
4. Center leadership training on meaning, mission, and inclusion. The 4P Framework’s emphasis on “Purpose” highlights the importance of values-based leadership. Programs should foster reflection, integrity, well-being, and connectedness. They should also recognize that effective leadership depends not only on skills, but also on motivation, meaning, and ethics.
5. Commit to ongoing evaluation and iterative improvement. All leadership development efforts should be accompanied by robust evaluation systems to measure impact, ensure responsiveness to emerging needs, and support continuous quality improvement.
6. Promote cross-sector collaboration and systems thinking. Leadership training should explicitly prepare public health professionals to work across boundaries - with healthcare, education, housing, business, and other sectors - reflecting the systems nature of public health challenges and the necessity of collaborative solutions.

By embedding these recommendations into educational and professional development infrastructures, we can cultivate a public health workforce that is not only technically proficient but also visionary, values-driven, and resilient. In an increasingly interconnected and uncertain world, such leadership will be the cornerstone of effective and equitable public health action.

## References

[1] Lachance JA Oxendine JS. Redefining leadership education in graduate public health programs: prioritization, focus, and guiding principles. Am J Public Health. (2015) 105:S60–4. doi: 10.2105/AJPH.2014.302463, PMID: 25706021 PMC4339997

[2] Beitsch LM Yeager VA Leider JP Erwin PC. Mass exodus of state health department deputies and senior management threatens institutional stability. Am J Public Health. (2019) 109:681–3. doi: 10.2105/ajph.2019.305005, PMID: 30969838 PMC6459643

[3] Hawley SR. Using adaptive leadership principles to support public health 3.0 in multidisciplinary undergraduate education. Leadersh Health Serv. (2021) 34:248–62. doi: 10.1108/lhs-07-2020-0051

[4] Rowitz L. Essentials of leadership in public health. Burlington, Ma: Jones & Bartlett Learning (2018).

[5] de Beaumont Foundation. Public health workforce interests and needs survey. de Beaumont Foundation. (2021). Available at: https://phwins.org/national

[6] Council on education for public health. Accreditation Criteria (2021). Available online at: https://media.ceph.org/documents/2021.Criteria.pdf

[7] Moodie R. Learning about self: leadership skills for public health. J Public Health Res. (2016) 5. doi: 10.4081/jphr.2016.679, PMID: 27190982 PMC4856874

[8] Koh HK Jacobson M. Fostering public health leadership. J Public Health. (2009) 31:199–201. doi: 10.1093/pubmed/fdp032 19451343

[9] Public Health Accreditation Board. (2025). Available online at: https://phaboard.org

[10] Reid WM Dold CJ. Leadership training and the problems of competency development. J Public Health Manag Pract. (2017) 23:73–80. doi: 10.1097/phh.0000000000000456, PMID: 27598708

[11] Jadhav ED Holsinger JW Anderson BW Homant N. Leadership for public health 3.0: a preliminary assessment of competencies for local health department leaders. Front Public Health. (2017) 5. doi: 10.3389/fpubh.2017.00272, PMID: 29085819 PMC5650692

[12] Begun JW Malcolm J. Leading public health: A competency framework. New York: Springer Publishing Company (2014).

[13] Council on Linkages between Academia and Public Health Practice. The council on linkages between academia and public health practice. (2014). Available online at: https://region2phtc.org/wp-content/uploads/2019/01/Core_Competencies_for_Public_Health_Professionals_2014June.pdf

[14] Grimm BL Watanabe-Galloway S Britigan DH Schumaker AM. A qualitative analysis to determine the domains and skills necessary to Lead in public health. J Leadersh Stud. (2015) 8:19–26. doi: 10.1002/jls.21342

[15] Public Health Accreditation Board. Standards & Measures for Initial Accreditation (2022). Available online at: https://phaboard.org/wp-content/uploads/Standards-Measures-Initial-Accreditation-Version-2022.pdf

[16] Turning Point Leadership Development National Excellence Collaborative. Leadership Development 113133 (2006) Available online at: http://216.92.113.133/Pages/leaddev.html. [Accessed April 25, 2025]

[17] National Board of Public Health Examiners. CPH job task analysis - NBPHE NBPHE (2024).

[18] Institute of Medicine. The future of public health. Washington, D.C.: National Academies Press (1988).

[19] Joan B. Kroc School of Peace Studies. What is a social innovator and how can I become one? (2023) Available online at: https://krocstories.sandiego.edu/peace/the-innovating-peace-blog/what-is-a-social-innovator-and-how-can-i-become-one [Accessed June 30, 2025]

[20] Battilana J Butler B Kimsey M Mair J Marquis C Seelos C. Problem, person, and pathway: a framework for social innovators. Social Innovation + Change Initiative (2018) Available online at: https://sici.hks.harvard.edu/3p-framework-for-social-innovators/

[21] Koh HK Tso CC Dougherty CP Lazowy EE Heberlein CP Phelps FA. Exploring the spiritual foundations of public health leadership. Front Public Health. (2023) 11. doi: 10.3389/fpubh.2023.1210160, PMID: 37954055 PMC10634334

[22] Council on Linkages Between Academia and Public Health Practice. Core competencies for public health professionals|PHF. PH|Advancing the public health workforce to achieve organizational excellence (2025) Available online at: https://phf.org/programs/core-competencies-for-public-health-professionals/

[23] Public Health Accreditation Board. The 10 essential public health services. Public health accreditation board (2020) Available online at: https://phaboard.org/center-for-innovation/public-health-frameworks/the-10-essential-public-health-services/

[24] Fry LW. Toward a theory of spiritual leadership. Leadersh Q. (2003) 14:693–727. doi: 10.1016/j.leaqua.2003.09.001

